# Models of heterogeneous dopamine signaling in an insect learning and memory center

**DOI:** 10.1371/journal.pcbi.1009205

**Published:** 2021-08-10

**Authors:** Linnie Jiang, Ashok Litwin-Kumar

**Affiliations:** 1 Mortimer B. Zuckerman Mind Brain Behavior Institute, Department of Neuroscience, Columbia University, New York, New York, United States of America; 2 Neurosciences Program, Stanford University, Stanford, California, United States of America; Research Center Jülich, GERMANY

## Abstract

The *Drosophila* mushroom body exhibits dopamine dependent synaptic plasticity that underlies the acquisition of associative memories. Recordings of dopamine neurons in this system have identified signals related to external reinforcement such as reward and punishment. However, other factors including locomotion, novelty, reward expectation, and internal state have also recently been shown to modulate dopamine neurons. This heterogeneity is at odds with typical modeling approaches in which these neurons are assumed to encode a global, scalar error signal. How is dopamine dependent plasticity coordinated in the presence of such heterogeneity? We develop a modeling approach that infers a pattern of dopamine activity sufficient to solve defined behavioral tasks, given architectural constraints informed by knowledge of mushroom body circuitry. Model dopamine neurons exhibit diverse tuning to task parameters while nonetheless producing coherent learned behaviors. Notably, reward prediction error emerges as a mode of population activity distributed across these neurons. Our results provide a mechanistic framework that accounts for the heterogeneity of dopamine activity during learning and behavior.

## Author summary

Dopamine neurons across the animal kingdom are involved in the formation of associative memories. While numerous studies have recorded activity in these neurons related to external and predicted rewards, the diversity of these neurons’ activity and their tuning to non-reward-related quantities such as novelty, movement, and internal state have proved challenging to account for in traditional modeling approaches. Using a well-characterized model system for learning and memory, the mushroom body of *Drosophila* fruit flies, Jiang and Litwin-Kumar provide an account of the diversity of signals across dopamine neurons. They show that models optimized to solve tasks like those encountered by flies exhibit heterogeneous activity across dopamine neurons, but nonetheless this activity is sufficient for the system to solve the tasks. The models will be useful to generate testable hypotheses about dopamine neuron activity across different experimental conditions.

## Introduction

Dopamine release modulates synaptic plasticity and learning across vertebrate and invertebrate species [1, 2]. A standard view of dopamine activity, proposed on the basis of recordings in the mammalian midbrain dopaminergic system, holds that dopamine neuron firing represents a “reward prediction error,” the difference between received and predicted reward [3]. This view is consistent with models of classical conditioning experiments and with reinforcement learning algorithms that learn to choose the most rewarding sequence of actions [4]. A frequent assumption in these models is that the scalar reward prediction signal is globally broadcast to and gates the modification of synaptic connections involved in learning. However, studies in both vertebrates and invertebrates suggest that dopamine neuron activity is modulated by other variables in addition to reward prediction error, and that this modulation is heterogeneous across populations of dopamine neurons [5].

**Fig 1:**
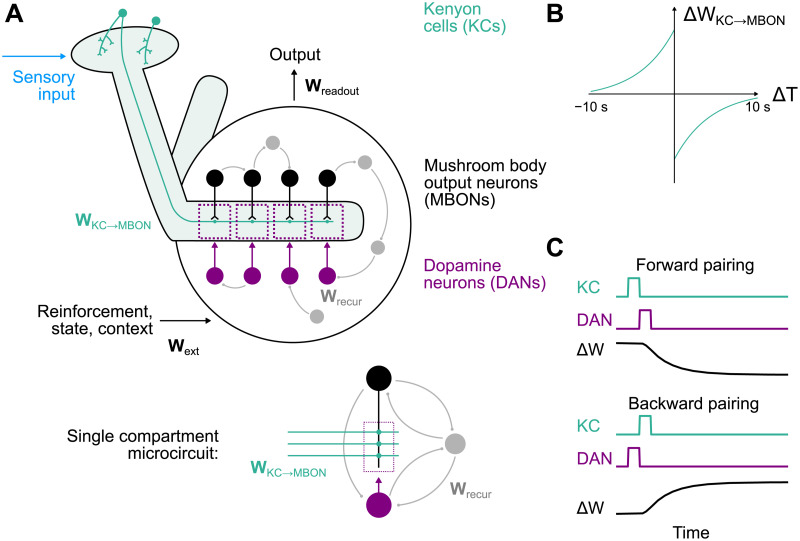
Diagram of the mushroom body model. **(A)** Kenyon cells (KCs) respond to stimuli and project to mushroom body output neurons (MBONs) via weights **W**_KC→MBON_. These connections are dynamic variables that are modified according to a synaptic plasticity rule gated by dopamine neurons (DANs). Output neurons and dopamine neurons are organized into compartments (dotted rectangles). External signals convey, e.g., reward, punishment, or context to the mushroom body output circuitry according to weights **W**_ext_. A linear readout with weights **W**_readout_ determines the behavioral output of the system. Connections among output neurons, dopamine neurons, and feedback neurons (gray) are determined by weights **W**_recur_. Inset: expanded diagram of connections in a single compartment. **(B)** The form of the dopamine neuron-gated synaptic plasticity rule operative at KC-to-MBON synapses. Δ*T* is the time difference between Kenyon cell activation and dopamine neuron activation. **(C)** Illustration of the change in KC-to-MBON synaptic weight Δ*W* following forward and backward pairings of Kenyon cell and dopamine neuron activity.

Early studies in arthropods identified roles for dopamine in a variety of functions [6–11]. In *Drosophila*, both memory [12] and other functions including locomotion, arousal, sleep, and mating have been associated with dopamine signaling [11]. Associative olfactory learning in *Drosophila* requires a central brain area known as the mushroom body [13–15], and many studies of dopamine neurons innervating this area have focused on activity related to reward and punishment and its roles in the formation of appetitive and aversive memories [16–22]. In the mushroom body, Kenyon cells (KCs, green neurons in Fig 1A) conveying sensory information, predominantly odor-related signals, send parallel fibers that contact the dendrites of mushroom body output neurons (MBONs, black neurons in Fig 1A). The activation of specific output neurons biases the organism toward particular actions [23, 24]. Output neuron dendrites define discrete anatomical regions, known as “compartments,” each of which is innervated by distinct classes of dopaminergic neurons (DANs, magenta neurons in Fig 1A). If the Kenyon cells and dopamine neurons that project to a given output neuron are both active within a particular time window, KC-to-MBON synapses are strengthened or weakened depending on the relative timing of Kenyon cell and dopamine neuron activation [25–28]. The resulting synaptic modifications permit flies to learn and update associations between stimuli and reinforcement.

In addition to classical reward and punishment signals, recent studies have shown that variables including novelty [29], reward prediction [30–32], and locomotion-related signals [33] are encoded by mushroom body dopamine neurons. In mammals, dopamine signals related to movement, novelty and salience, and separate pathways for rewards and punishment have also been identified in midbrain regions [5, 34–42]. These observations call for extensions of classic models that assume dopamine neurons in associative learning centers are globally tuned to reward prediction error [43]. How can dopamine signals gate appropriate synaptic plasticity and learning if their responses are modulated by mixed sources of information?

To address this question, we develop a modeling approach in which networks that produce dopamine signals suited to learning a particular set of behavioral tasks are constructed. Our key methodological advance is to augment standard recurrent neural network models, which employ fixed synaptic weights to solve tasks after optimization [44], with synapses that exhibit fast dopamine-gated plasticity via an experimentally determined plasticity rule [28]. We employ a “meta-learning” approach involving two phases [45–47]. First, we optimize the network connections responsible for producing suitable learning signals in dopamine neurons. Next, after these connections are fixed, we examine the network’s behavior on novel tasks in which learning occurs only via biologically plausible dopamine-gated plasticity. Due to the well-characterized anatomy of the mushroom body and knowledge of this plasticity rule, our approach allows us to generate predictions about the activity of multiple neuron types [28, 48]. Comprehensive synapse-level wiring diagrams for the output circuitry of the mushroom body have recently become available, which will allow the connectivity of models constructed with our approach to be further constrained by data [49–53]. As the dynamics of our models, including the dopamine-gated plasticity, are optimized end-to-end only for overall task performance, our model predictions do not require a priori assumptions on what signals the dopamine neurons encode. In particular, our methods do not assume that each dopamine neuron carries a reward prediction error and instead allow for heterogeneous activity across the population.

The meta-learned networks we construct are capable of solving complex behavioral tasks and generalizing to novel stimuli using only experimentally constrained plasticity rules, as opposed to networks that require gradient descent updates to network parameters to generalize to new tasks. They can form associations based on limited numbers of stimulus/reinforcement pairings and are capable of continual learning, which are often challenging for artificial neural networks [46, 54]. In the models, different dopamine neurons exhibit diverse tuning to task-related variables, while reward prediction error emerges as a mode of activity across the population. Our approach uncovers the mechanisms behind the observed heterogeneity of dopamine signals in the mushroom body and suggests that the “error” signals that support associative learning may be more distributed than is often assumed.

## Results

### Modeling recurrent mushroom body output circuitry

The diversity of dopamine neuron activity challenges models of mushroom body learning that assume these neurons convey global reward or punishment signals. Part of this discrepancy is likely due to the intricate connectivity among output neurons, dopamine neurons, and other neurons that form synapses with them [48, 52, 53]. We therefore modeled these neurons and their connections, which we refer to collectively as the mushroom body “output circuitry,” as a recurrent neural network (Fig 1A). This model network consists of 20 output neurons, 20 dopamine neurons, and 60 additional recurrent feedback neurons. Recurrent connections within the network are defined by a matrix of synaptic weights **W**_recur_. Connections between all of these 100 neurons are permitted, except that we assume connections from dopamine neurons to output neurons are modulatory and follow a compartmentalized organization (Fig 1A, inset). Synapses from 200 Kenyon cells onto output neurons provide the network with sensory information and are represented by **W**_KC→MBON_. Separate pathways convey signals such as reward or punishment from other brain regions, via weights **W**_ext_.

The dynamics of the *i*th neuron in our model of the output circuitry are given by:

$$\begin{array}{c} \tau \frac{dr_{i}\left(t\right)}{dt}=-r_{i}\left(t\right)+{\left[\sum_{j}W_{ij}^{\text{recur}}r_{j}\left(t\right)+b_{i}+I_{i}\left(t\right)\right]}_{+}, \end{array}$$ (1)

where [⋅]_+_ represents positive rectification. The bias *b*_*i*_ determines the excitability of neuron *i*, while *I*_*i*_(*t*) represents its input from non-recurrent connections. If neuron *i* is an output neuron, then its external input is given by $I_{i}\left(t\right)=\sum_{k}W_{ik}^{\text{KC}\to \text{MBON}}\left(t\right)r_{k}^{\text{KC}}\left(t\right)$, representing input from Kenyon cells. If neuron *i* is a feedback neuron (FBN), then $I_{i}\left(t\right)=\sum_{k}W_{ik}^{\text{ext}}r_{k}^{\text{ext}}\left(t\right)$, representing reinforcement, context, or state-dependent input from other brain regions. For dopamine neurons, *I*_*i*_(*t*) = 0, as all input to the dopamine neurons is relayed by feedback neurons, reflecting our interpretation of the feedback neuron population as containing any pathway that conveys information to the dopamine neurons. We do not constrain **W**^recur^, except that entries corresponding to connections from dopamine neurons to output neurons are set to zero, based on the assumption that these connections modulate plasticity of KC-to-MBON synapses rather than output neuron firing directly (but see [50] and Discussion).

The objective of the network is to generate a desired pattern of activity in a readout that represents the behavioral bias produced by the mushroom body. The readout decodes this desired output through a matrix of weights **W**_readout_. In our first set of experiments, this readout will represent the one-dimensional valence (appetitive vs. aversive) of a stimulus decoded from the output neurons (meaning that **W**_readout_ is a 1 × *N*_MBON_ matrix; later, we will consider more sophisticated readouts):

$$\begin{array}{c} v\left(t\right)=W_{\text{readout}}r_{\text{MBON}}\left(t\right). \end{array}$$ (2)

To achieve the task goal, trials are randomly generated and the following objective function, which depends on the parameters of the network *θ* and represents the loss corresponding to an individual trial consisting of *T* discretized timesteps {*t*_1_, *t*_2_, … *t*_*T*_}, is minimized through stochastic gradient descent:

$$\begin{array}{c} L_{\theta }=\frac{1}{T}\sum_{n=1}^{T}{\left(v\left(t_{n}\right)-v^{*}\left(t_{n}\right)\right)}^{2}+\frac{\lambda }{T}\sum_{n=1}^{T}\sum_{i=1}^{N_{\text{DAN}}}{\left[r_{i}^{\text{DAN}}\left(t_{n}\right)-0.1\right]}_{+}^{2}. \end{array}$$ (3)

The first term represents the difference between the decoded valence and a target valence v* that is determined by the task being learned. The second term is a regularization term that penalizes dopamine neuron activity that exceeds a baseline level of 0.1 (in normalized units of firing rate and with *λ* = 0.1). This term was included to promote solutions that do not exhibit high levels of non-task-related dopamine activity, but we verified with simulations that the regularization does not significantly affect overall network performance. Example loss curves over the course of network optimization are shown in S1 Fig.

### Implementation of dopamine-gated plasticity

Recurrent network modeling approaches typically optimize all parameters *θ* of the network in order to produce a desired behavior. This approach assumes that, after optimization, connections are fixed to constant values during the execution of the behavior. However, connections between Kenyon cells and output neurons are known to exhibit powerful and rapid dopamine-gated synaptic plasticity. This plasticity is dependent on the relative timing of Kenyon cell and dopamine neuron activation (notably, it does not appear to depend on the postsynaptic output neuron firing rate [26]) and can drive substantial changes in evoked output neuron activity even after brief KC-DAN pairings [28]. We therefore augmented our networks with a model of this plasticity by assuming that each element of **W**_KC→MBON_ is a dynamic quantity that tracks the variables *w*_*ij*_ (with a time constant of *τ*_*W*_ = 5 s; see below). These variables, which determine the strength of the connection from the *j*th Kenyon cell to the *i*th output neuron, obey the following update rule:

$$\begin{array}{c} \frac{dw_{ij}\left(t\right)}{dt}={\bar{r}}_{i}^{\text{DAN}}\left(t\right)r_{j}^{\text{KC}}\left(t\right)-{\bar{r}}_{j}^{\text{KC}}\left(t\right)r_{i}^{\text{DAN}}\left(t\right), \end{array}$$ (4)

where $r_{j}^{\text{KC}}$ and $r_{i}^{\text{DAN}}$ are the firing rates of the *j*th Kenyon cell and the dopamine neuron that innervates the *i*th compartment, and ${\bar{r}}_{j}^{\text{KC}}$ and ${\bar{r}}_{i}^{\text{DAN}}$ are synaptic eligibility traces constructed by low-pass filtering $r_{j}^{\text{KC}}$ and $r_{i}^{\text{DAN}}$. The time constants of the low-pass filters used to generate the eligibility traces determine the time window within which pairings of Kenyon cell and dopamine neuron activity elicit appreciable changes of *w*. Each weight element of **W**_KC→MBON_ is initially set to its maximum value of 0.05 and subsequently updated according to $\tau_{W}\frac{dW_{ij}^{\text{KC}\to \text{MBON}}\left(t\right)}{dt}=-W_{ij}^{\text{KC}\to \text{MBON}}\left(t\right)+w_{ij}\left(t\right)$. The timescale of *τ*_*W*_ = 5 s accounts for the timescale of the induction of LTD or LTP.

Odors are encoded by sparse activation of random subsets of Kenyon cells, which is accomplished in the model by setting 10% of the elements of **r**_KC_ to 1 and the rest to 0. When Kenyon cell and dopamine neuron firing rates are modeled as pulses separated by a time lag Δ*T*, the dependence of the change in *w*_*ij*_ on Δ*T* takes the form of a biphasic timing-dependent function (Fig 1B and 1C), consistent with a recent experimental characterization [28]. The seconds-long timescale of this curve is compatible with the use of continuous firing rates rather than discrete spike timing to model KC-to-MBON plasticity, as we have done in Eq 4.

**Fig 2:**
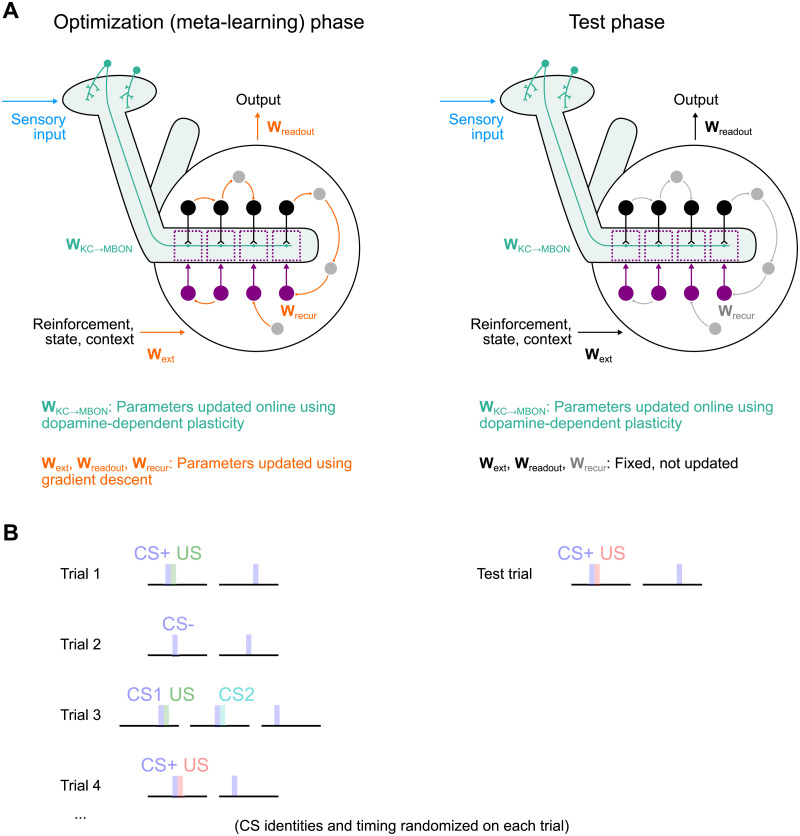
Schematic of meta-learning procedure. **(A)** Two phases of meta-learning and testing. Left: During the optimization phase, connections that form the mushroom body output circuitry are updated with gradient descent (orange). Kenyon cell to output neuron weights evolve “online” (within each trial) according to dopamine-dependent synaptic plasticity. Right: After optimization is complete, the network is tested on a new set of trials. In this phase, connections that form the output circuitry are fixed. **(B)** Illustration of trials involving CS/US associations presented during training (left) and testing (right). Each trial involves new CS/US identities and timing.

Importantly, the weight update rule in Eq 4 is a smooth function of network firing rates, allowing networks with this update rule to be constructed using gradient descent. Specifically, we minimize the loss function Eq 3 under the assumption that the network follows the dynamics defined by Eqs 1 and 4. The parameters to be optimized are *θ* = {**W**_recur_, **W**_ext_, **W**_readout_, **b**} (the connections describing the mushroom body output circuitry and the biases), while **W**_KC→MBON_ is treated as a dynamic quantity. We refer to the gradient descent modification of *θ* as the “optimization” phase of constructing our networks. This optimization represents the evolutionary and developmental processes that produce a network capable of efficiently learning new associations [55]. After this optimization is complete, the output circuitry is fixed, but KC-to-MBON weights are subject to synaptic plasticity according to Eq 4. Our approach therefore separates synaptic weight changes that are the outcome of evolution and development from those due to experience-dependent KC-to-MBON plasticity, which would be conflated if all parameters were optimized with gradient descent (Fig 2). We show that, after optimization, only the latter form of biologically plausible weight update is sufficient to solve the tasks we consider and generalize to related but new tasks.

To begin, we assume that KC-to-MBON weights are set to their baseline values at the beginning of each trial in which new associations are formed. Later, we will consider the case of continual learning of many associations.

### Models of associative conditioning

We begin by considering models of classical conditioning, which involve the formation of associations between a conditioned stimulus (CS) and unconditioned stimulus (US) such as reward or punishment. A one-dimensional readout of the output neuron population is taken to represent the stimulus valence (Eq 2), which measures whether the organism prefers (valence > 0) or avoids (valence < 0) the CS. In the model, CS are encoded by the activation of a random ensembles of Kenyon cells. Rewards and punishments are encoded by external inputs to the network that provide input through **W**_ext_ (see Methods).

**Fig 3:**
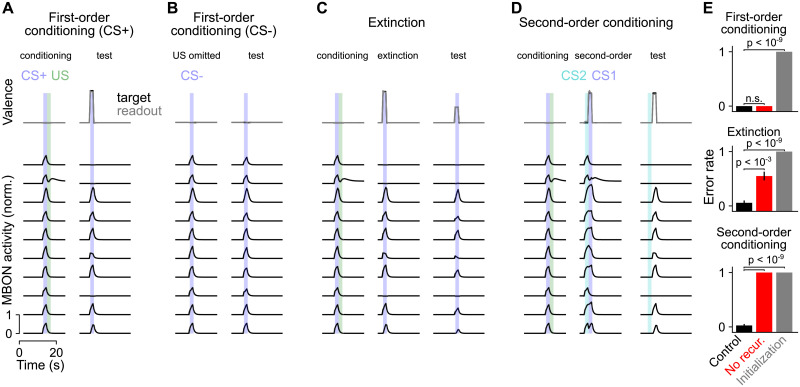
Behavior of network during reward conditioning paradigms. **(A)** Behavior of output neurons (MBONs) during first-order conditioning. During training, a CS+ (blue) is presented, followed by a US (green). Top: The network is optimized so that a readout of the output neuron activity during the second CS+ presentation encodes valence (gray curve). Black curve represents the target valence and overlaps with the readout. Bottom: Example responses of output neurons. **(B)** Same as **A**, but for CS- presentation without US. **(C)** Same as **A**, but for extinction, in which a second presentation of the CS+ without the US partially extinguishes the association. **(D)** Same as **A**, but for second-order conditioning, in which a second stimulus (CS2) is paired with a conditioned stimulus (CS1). **(E)** Error rate averaged across networks in different paradigms. An error is defined as a difference between reported and target valence with magnitude greater than 0.2 during the test period. Networks optimized with recurrent output circuitry (control, black) are compared to networks without recurrence (no recur., red), and networks prior to optimization (initialization, gray). Error rates for each network realization are evaluated over 50 test trials and used to generate *p*-values with a Mann-Whitney *U*-test over 20 network realizations.

To construct the model, we optimized the mushroom body output circuitry to produce an estimate of the target valence in the readout during presentation of CS+ that have been paired with US (first-order conditioning; Fig 3A and 3B, top). During presentations of novel CS-US pairings after optimization, this valence is reported for CS+ but not unconditioned stimulus (CS-) presentations. The activities of subsets of model output neurons are suppressed following conditioning, indicating that the network learns to modify its responses for CS+ but not CS- responses (Fig 3A and 3B, bottom). This form of classical conditioning requires an appropriate mapping from US pathways to dopamine neurons, but recurrent mushroom body output circuitry is not required; networks without recurrence also produce the target valence (Fig 3E; top). We therefore considered a more complex set of tasks. Networks were optimized to perform first-order conditioning, to extinguish associations upon repeated presentation of a CS+ without US, and also to perform second-order conditioning.

During extinction, the omission of a US following a previously conditioned CS+ reduces the strength of the learned association (Fig 3C). In second-order conditioning, a CS (CS1) is first paired with a reward or punishment (Fig 3D, left), and then a second CS (CS2) is paired with CS1 (Fig 3D, center). Because CS2 now predicts CS1 which in turn predicts reward or punishment, the learned valence of CS1 is transferred to CS2 (Fig 3D, right). In both extinction and second-order conditioning, a previously learned association must be used to instruct either the modification of an existing association (in the case of extinction) or the formation of a new association (in the case of second-order conditioning). We hypothesized that recurrent output circuitry would be required in these cases. Indeed, non-recurrent mushroom body networks are unable to solve these tasks, while recurrent networks are (Fig 3E, center, bottom). Non-recurrent networks optimized for multiple tasks also exhibited errors on first-order conditioning (0.0 and 0.42 error rate for recurrent and non-recurrent networks respectively, *p* < 10^−8^, Mann-Whitney *U*-test), indicating a general failure to optimize. Recurrent networks generalized to related tasks that they were not optimized for, such as reversal learning (S2 Fig), further supporting the conclusion that they implement generalizable learning strategies.

We also examined whether the addition of direct connections from Kenyon cells to dopamine neurons influenced our results (S3 Fig). Such connections are present across mushroom body compartments, but their functional properties are unclear [49, 56]. Our qualitative results were unchanged when these connections were added, whether we assumed they were fixed or subject to synaptic plasticity. Thus, indirect connections from Kenyon cells to dopamine neurons through recurrent mushroom body circuitry are sufficient for the tasks we consider.

### Comparison to networks without plasticity

Standard recurrent neural networks can maintain stimulus information over time through persistent neural activity, without modification of synaptic weights. This raises the question of whether the dopamine-gated plasticity we implemented is necessary to recall CS-US associations, or if recurrent mushroom body output circuitry alone is sufficient. We therefore compared the networks described above to networks lacking this plasticity. For non-plastic networks, connections from Kenyon cells to output neurons are optimized through gradient descent (with no constraints on excitatory or inhibitory sign) and fixed after optimization. These networks evolve similarly to plastic networks except that the dynamics are determined only by Eq 1 and not by the dopamine-gated plasticity of Eq 4.

**Fig 4:**
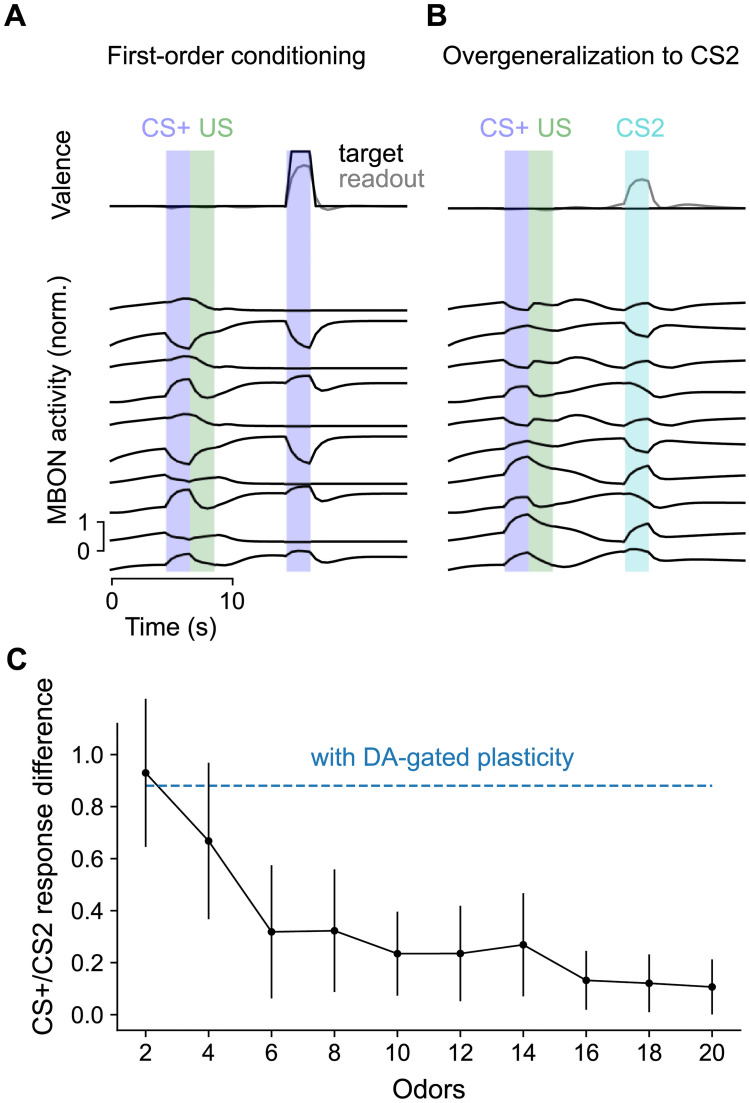
Comparison to networks without dopamine-gated plasticity. **(A)** Behavior during first-order conditioning, similar to Fig 3A, but for a non-plastic network. Because of the need for non-plastic networks to maintain information using persistent activity, performance degrades with longer delays between training and test phases. We therefore chose this delay to be shorter than in Fig 3A. Results are shown for a network optimized with 10 odors. **(B)** Same as **A**, but for a trial in which a CS-US pairing is followed by the presentation of a neutral CS. **(C)** Difference in response (reported valence) for CS+ and neutral CS as a function of the number of odors. Each CS+ is associated with either a positive or negative US. For comparison, the corresponding response difference for networks with dopamine-gated plasticity is shown in blue. Error bars represent s.e.m. over 8 network realizations.

In non-plastic networks, information about CS-US associations must be stored in the persistent activity of the mushroom body output circuitry as an “attractor” of neural activity [57], as opposed to being encoded in the KC-to-MBON weights. Such activity must maintain both the identity of the CS+ odor and which US it was paired with in order to recall the learned valence without generalizing that valence to a different odor. We hypothesized that it would be challenging for networks to support a large number of such attractors and therefore investigated the performance of non-plastic networks in simulated environments in which there are a fixed number of odors. We optimized networks both to respond appropriately to CS+ (Fig 4A) and avoid responding to neutral CS (Fig 4B).

Non-plastic networks can form CS-US associations (Fig 4A). Compared to networks with dopamine-gated plasticity (Fig 3A), output neurons exhibit stronger persistent activity following a CS-US pairing. However, when the number of odors in the environment is large, non-plastic networks exhibit a high degree of overgeneralization of learned associations to neutral CS that have not been paired with US (Fig 4B). This likely reflects the non-plastic networks’ inability to distinguish between odor identities when many odors are present. When odor identities cannot be distinguished, the best compromise is to assume that the learned CS+ valence applies to both the CS+ and to neutral CS, and indeed when many odors are present the difference in the reported valence for these two classes of stimuli decreases to zero (Fig 4C). Networks with dopamine-gated plasticity do not suffer from this shortcoming, as they can store and update the identities of arbitrary novel stimuli in KC-to-MBON weights (Fig 4C, blue curve).

In total, the comparison between plastic and non-plastic networks demonstrates that the addition of dopamine-gated plasticity at KC-to-MBON synapses improves capacity and reduces overgeneralization. Furthermore, plastic networks need not rely solely on persistent activity in order to store associations (compare Figs 3A and 4A), likely prolonging the timescale over which information can be stored without being disrupted by ongoing activity.

### Distributed representations across dopamine neurons

**Fig 5:**
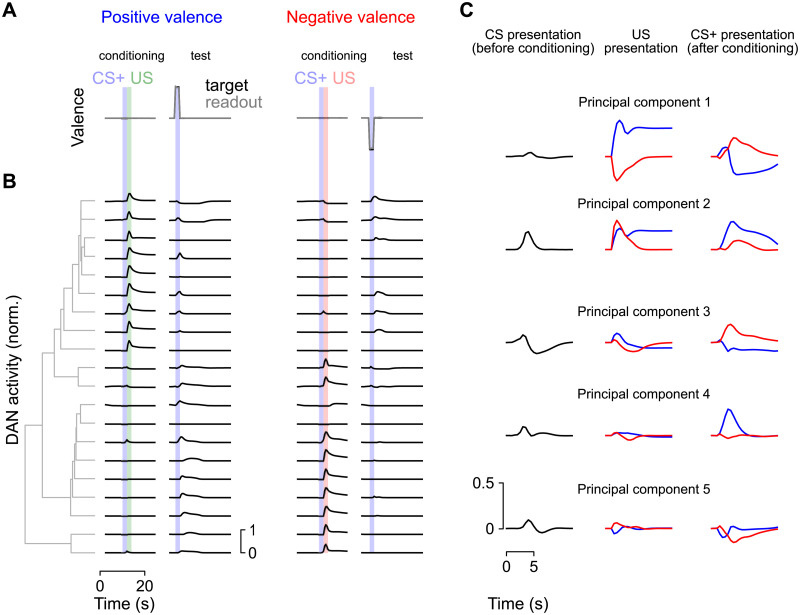
Population analysis of dopamine neuron (DAN) activity. **(A)** First-order conditioning trials with positive or negative valence US. **(B)** Responses of model dopamine neurons from a single network. Neurons are sorted according to hierarchical clustering (illustrated with gray dendrogram) of their responses. **(C)** Principal components analysis of dopamine neuron population activity. Left: Response to CS before conditioning. Middle: Response to a positive (green) or negative (red) valence US. Right: Response to a previously conditioned CS.

We next examined the responses of dopamine neurons to neutral, unconditioned, and conditioned stimuli in the networks we constructed, to examine the “error” signals responsible for learning (Fig 5A). Dopamine neurons exhibited heterogeneity in their responses. We performed hierarchical clustering to identify groups of dopamine neurons with similar response properties (Fig 5B, gray; see Methods). This procedure identified two broad groups of dopamine neurons—one that responds to positive-valence US and another that responds to negative-valence US—as well as more subtle features in the population response. Consistent with the known logic of the mushroom body output circuitry [48] and learning involving depression of KC-to-MBON synapses, compartments whose dopamine neurons signal positive valence US tend to have output neurons whose activation signals negative valence (S4 Fig).

While some dopamine neurons increase their firing only for US, many also respond to reinforced CS. In some cases, this response includes a decrease in firing rate in response to the omission of a predicted US that would otherwise cause an increase in rate, consistent with a reward prediction error. In other cases, neurons respond only with increases in firing rate for US of a particular valence, and for omitted US of the opposite valence, consistent with cross-compartmental interactions supporting the prediction of valence [31]. The presence of both reward prediction error-like responses and valence-specific omission responses suggests that multiple mechanisms are employed by the network to perform tasks such as extinction and second-order conditioning.

The examination of their responses demonstrates that dopamine neurons in our models are diversely tuned. This tuning implies that KC-to-MBON synapses change in a heterogeneous manner in response to CS and US presentations, but that these changes are sufficient to produce an appropriate behavioral response collectively. Consistent with this idea, principal components analysis of dopamine neuron responses identified modes of activity with interpretable, task-relevant dynamics. The first principal component (Fig 5C) reflects US valence and predicted CS+ valence, while rapidly changing sign upon US omission, consistent with a reward prediction error. It is notable that such a signal emerges as the dominant mode of dopamine neuron activity, as our optimization procedure does not explicitly require the formation of a reward prediction error. Subsequent principal components include components that respond to CS and US of both valences (principal component 2) or are tuned primarily to a single stimulus, such as a positive valence CS+ (principal component 4). When we constrained networks to have fewer compartments, error increased (S5 Fig) suggesting that diversity in dopamine signaling improves performance, though we note that this trend does not distinguish task difficulty and ease of optimization.

**Fig 6:**
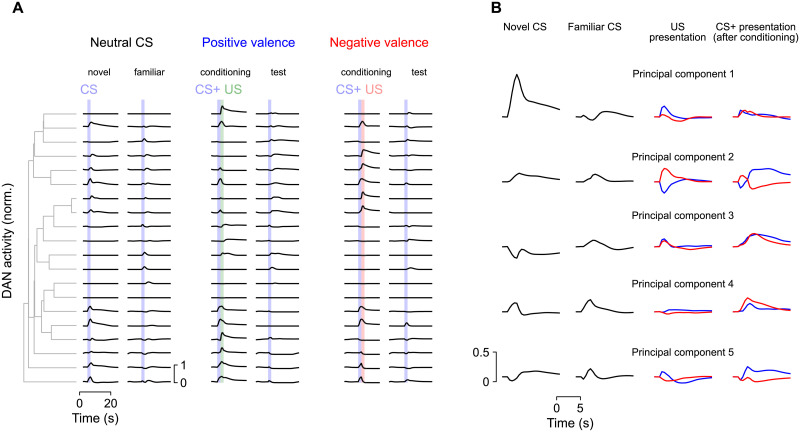
Behavior of a network that encodes both valence and novelty. The network is similar to Fig 5 but a second readout that computes novelty is added. The novelty readout is active for the first presentation of a given CS and zero otherwise. **(A)** The addition of novelty as a readout dimension introduces dopamine neuron responses that are selective for novel CS. Compare with Fig 5B. **(B)** The first principal component (PC1) for the network in **A** is selective for CS novelty. Compare with Fig 5C.

To further explore how dopamine neuron responses depend on the task being learned, we extended the model to require encoding of novelty and familiarity, inspired by a recent study that showed that the mushroom body is required for learning and expressing an alerting behavior driven by novel CS [29]. We added a second readout that reports CS novelty, in addition to the readout of valence described previously. Networks optimized to report both variables exhibit enhanced CS responses and a large novelty-selective component in the population response identified by principal components analysis (Fig 6), compared to networks that only report valence (Fig 5B). These results suggest that dopamine neurons collectively respond to any variables relevant to the task for which the output circuitry is optimized, which may include variables distinct from reward prediction. Furthermore, the distributed nature of this representation implies that individual variables may be more readily decoded from populations of dopamine neurons than from single neurons.

### Continual learning of associations

In the previous sections, we modeled the dynamics of networks during individual trials containing a limited number of associations. We next ask whether these networks are capable of continual learning, in which long sequences of associations are formed, with recent associations potentially overwriting older ones. Such learning is often challenging, particularly when synaptic weights have a bounded range, due to the tendency of weights to saturate at their minimum or maximum value after many associations are formed [58]. To combat this, a homeostasic process that prevents such saturation is typically required. We therefore asked if our optimized networks can implement such homeostasis.

**Fig 7:**
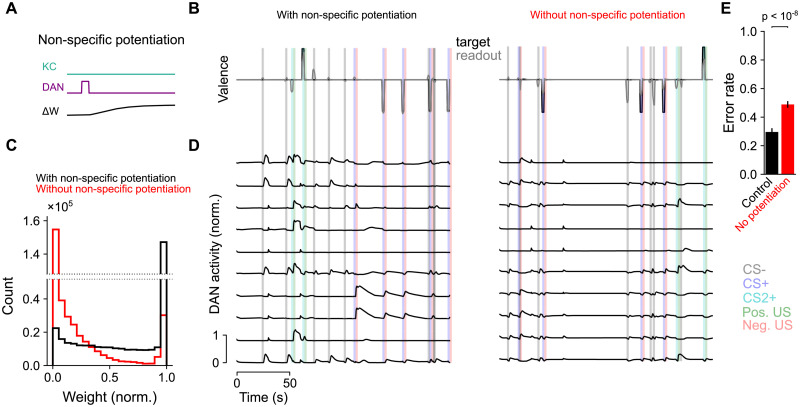
Model behavior for long sequences of associations. **(A)** Illustration of non-specific potentiation following dopamine neuron activity (compare with Fig 1C). **(B)** Example sequence of positive and negative associations between two odors CS+ and CS2+ and US. Neutral gray odors (CS-) are also presented randomly. **(C)** Histogram of synaptic weights after a long sequence of CS and US presentations for networks with (black) and without (red) non-specific potentiation. Weights are normalized to their maximum value. The means of the distributions across 18 network realizations for each condition were significantly different (*p* < 2 ⋅ 10^−7^, Mann-Whitney *U*-test). **(D)** Left: dopamine neuron responses for the sequence of CS and US presentations. Right: same as left, but for a network without non-specific potentiation. **(E)** Error rate (defined as a difference between reported and target valence with magnitude greater than 0.5 during a CS presentation; we used a higher threshold than Fig 3 due to the increased difficulty of the continual learning task) for networks with (black) and without (red) non-specific potentiation. Error rates for each network realization are evaluated over 20 test trials and used to generate *p*-values with a Mann-Whitney *U*-test over 18 network realizations.

In certain compartments of the mushroom body, it has been shown that the activation of dopamine neurons in the absence of Kenyon cell activity leads to potentiation of KC-to-MBON synapses [33]. This provides a mechanism for the erasure of memories formed following synaptic depression. We hypothesized that this non-specific potentiation could implement a form of homeostasis that prevents widespread synaptic depression after many associations are formed. We therefore augmented our dopamine-gated synaptic plasticity rule (Fig 1C) with such potentiation (Fig 7A). The new synaptic plasticity rule is given by:

$$\begin{array}{c} \frac{dw_{ij}\left(t\right)}{dt}={\bar{r}}_{i}^{\text{DAN}}\left(t\right)r_{j}^{\text{KC}}\left(t\right)-{\bar{r}}_{j}^{\text{KC}}\left(t\right)r_{i}^{\text{DAN}}\left(t\right)+\beta {\bar{r}}_{i}^{\text{DAN}}\left(t\right), \end{array}$$ (5)

where *β* represents the rate of non-specific potentiation (compare with Eq 4). We allowed *β* to be optimized by gradient descent individually for each compartment but constrained it to be nonnegative.

We modeled long sequences of associations in which CS+, CS-, and US are presented randomly (Fig 7B) and the network is again optimized to produce a target valence (Eq 3). In optimized networks, the KC-to-MBON weights are initialized at the beginning (*t* = 0) of trial *n* to be equal to those at the end (*t* = *T*) of trial *n* − 1, $W_{n}^{\text{KC}\to \text{MBON}}\left(0\right)=W_{n-1}^{\text{KC}\to \text{MBON}}\left(T\right)$, rather than being reset to their baseline values as done previously. We examined the distribution of KC-to-MBON synaptic weights after such sequences of trials. Without non-specific potentiation, most synaptic weights are clustered near 0 (Fig 7C, red). However, the addition of this potentiation substantially changes the synaptic weight distribution, with many weights remaining potentiated even after thousands of CS and US presentations (Fig 7C, black). We also examined performance and dopamine neuron responses in the two types of networks. Without non-specific potentiation, dopamine neuron responses are weaker and the reported valence less accurately tracks the target valence, compared to networks with such potentiation (Fig 7D and 7E).

These results suggest that such homeostatic mechanisms, or other modifications to the synaptic plasticity rule in Eq 4 that avoid weights clustering near 0, are important for performance on continual learning tasks. However, we note that non-specific potentiation might also shorten memory lifetime, for example in a situation where a CS-US pairing is followed by unpaired US presentations. Investigating how this tradeoff is resolved across compartments is an interesting topic for future study.

### Associating stimuli with changes in internal state

In the previous sections, we focused on networks whose dopamine neurons exhibited transient responses to the presentation of relevant external cues. Recent studies have found that dopamine neurons also exhibit continuous fluctuations that track the state of the fly, even in the absence of overt external reinforcement. These fluctuations are correlated with transitions between, for example, movement and quiescence [33], or hunger and satiation [59]. Understanding the functional role of this activity is a major challenge for models of dopamine-dependent learning. We hypothesized that such activity could permit the association of stimuli with a transition to an arbitrary internal state of the organism. This could allow downstream networks to read out whether a stimulus has previously been experienced in conjuction with a particular change in state, which might inform an appropriate behavioral response to that stimulus.

**Fig 8:**
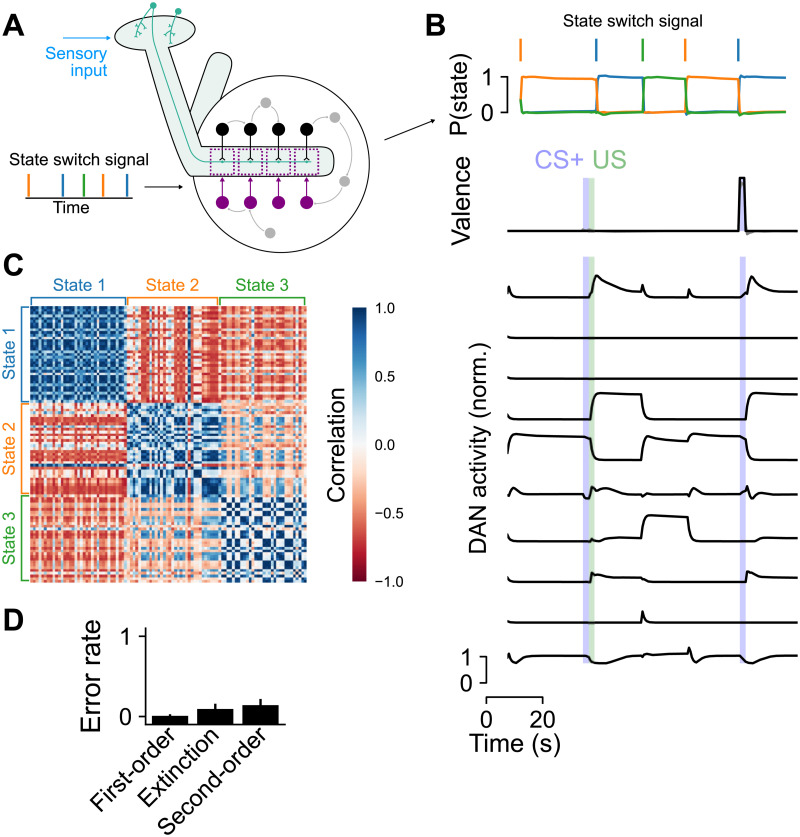
Behavior of a network whose activity transitions between a sequence of discrete states in addition to supporting associative conditioning. **(A)** Brief pulse inputs to the network signal that a switch to a new state should occur. **(B)** Top: A linear readout of dopamine neuron activity can be used to decode the network state. Bottom: dopamine neuron (DAN) activity exhibits state-dependent fluctuations in addition to responding to CS and US. **(C)** Decoding of stimuli that predict state transitions. Heatmap illustrates the correlation between output neuron population responses to the presentation of different stimuli that had previously been presented prior to a state transition. Stimuli are ordered based on the state transitions that follow their first presentation. Blue blocks indicate that stimuli that predict the same state transition evoke similar output neuron activity. **(D)** Performance of networks on conditioning tasks. For each network realization, error rates are computed over 50 test trials and bars represent s.e.m. over 40 network realizations.

To test this hypothesis, we constructed networks that, in addition to supporting associative conditioning (as in Fig 3), also transitioned between a set of three discrete internal states, triggered on input pulses that signal the identity of the next state (Fig 8A). This input represents signals from other brain areas that drive state transitions. We optimized the output circuitry to, in addition to encoding valence as before, continuously maintain a state representation, quantified by the ability of a linear readout of dopamine neuron activity to decode the current state (Fig 8B, top). Specifically, the loss function equaled

$$\begin{array}{c} L_{\theta }=\frac{1}{T}\sum_{n=1}^{T}{\left(v\left(t_{n}\right)-v^{*}\left(t_{n}\right)\right)}^{2}+\frac{1}{T}\sum_{n=1}^{T}||s\left(t_{n}\right)-s^{*}\left(t_{n}\right){\left|\right|}^{2}, \end{array}$$ (6)

where **s** = Softmax(**W**_stater_ **r**_DAN_) is a 3-dimensional vector that represents the decoded probabilities of being in each state and s* is a vector with one nonzero entry corresponding to the actual current state. Here, **W**_state_ is a 3 × *N*_DAN_ matrix of weights that represents a linear readout of the state from DANs, while as before valence is read out from MBONs. Because we were interested in networks that exhibited continuous fluctuations in dopamine neuron activity, we did not impose an additional penalty on dopamine neuron firing rates as in Eq 3. Optimizing networks with this loss function led to widespread state-dependent activity throughout the network, including among dopamine neurons (Fig 8B, bottom). This activity coexists with activity evoked by CS or US presentation.

We next examined output neuron responses to the presentation of stimuli that had previously preceded a transition to some state. If a transition to a given state reliably evokes a particular pattern of dopamine neuron activity, then KC-to-MBON synapses that are activated by any stimulus preceding such a transition will experience a similar pattern of depression or potentiation. We assessed this response similarity by computing the Pearson’s correlation coefficient $\text{Corr}(r_{A}^{\text{MBON}},r_{B}^{\text{MBON}})$, where $r_{A}^{\text{MBON}}$ is the average output neuron activity during the presentation of stimulus *A*. Consistent with this prediction, the pattern of output neuron responses evoked by a stimulus that predicts a transition to state *S*_1_ is more similar to the corresponding responses to other stimuli that predict the same state than any other state *S*_2_ (Fig 8C). The representations of state-transition-predictive stimuli are thus “imprinted” with the identity of the predicted state. While these modifications could potentially interfere with the ability of the system to support associative conditioning, these networks still exhibited high performance on the tasks we previously considered (Fig 8D). Thus, state-dependent activity and activity required for conditioning are multiplexed in the network. The presence of state-dependent fluctuations could allow circuits downstream of the mushroom body to consistently produce a desired behavior that depends on the internal state, instead of or in addition to the external reinforcement, that is predicted by a stimulus. Our model thus provides a hypothesis for the functional role of state-dependent dopamine neuron activity.

### Mixed encoding of reward and movement in models of navigation

**Fig 9:**
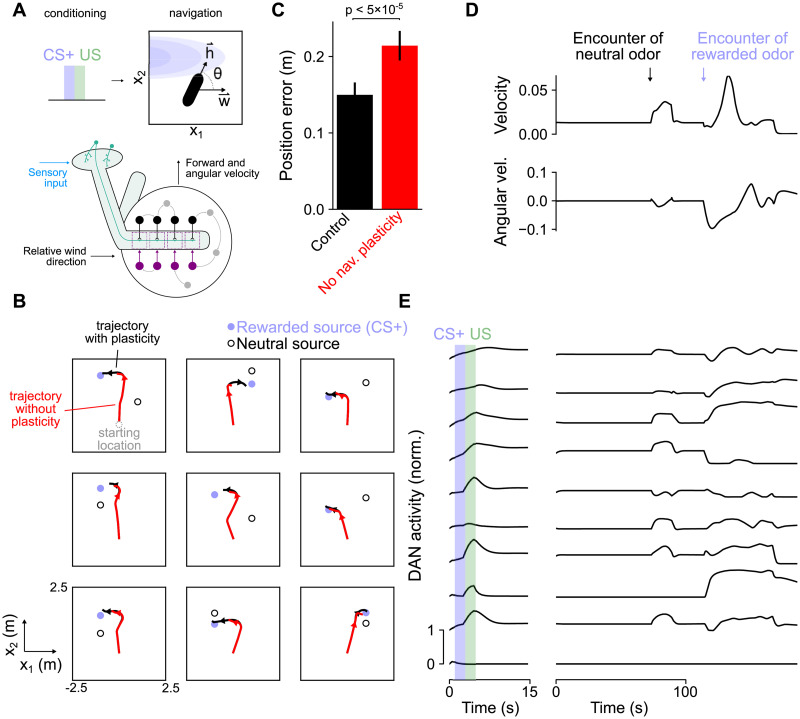
Model behavior for a navigation task. **(A)** Top: Schematic of navigation task. After conditioning, the simulated organism uses odor concentration input (blue) and information about wind direction **w** relative to its heading **h**. Bottom: Diagram of a network that uses these signals to compute forward and angular velocity signals for navigation. Velocity signals are read out from other neurons in the mushroom body output circuitry (gray), rather than output neurons. **(B)** Position of the simulated organism as a function of time during navigation. Black: Simulation with intact dopamine-gated plasticity during navigation; Red: Simulation with plasticity blocked. Arrowheads indicate direction of movement. In the top left plot, the starting location (gray circle) is indicated. **(C)** Position error (mean-squared distance from rewarded odor source at the end of navigation) for control networks and the same networks in which dopamine-gated plasticity is blocked during the navigation phase. For each network realization, error rates are computed over 50 test trials and bars represent s.e.m. over 30 network realizations. Significance is computed with a Wilcoxon signed-rank test. **(D)** Forward (top) and angular (bottom) velocity as a function of time during one example navigation trial. **(E)** Left: Dopamine neuron activity during CS and US presentation in the conditioning phase of a trial. Right: Dopamine neuron activity during the navigation phase of the trial (same trial as in **D**).

We also examined models of dynamic, goal directed behaviors. An important function of olfactory associations in *Drosophila* is to enable navigation to the sources of reward-predicting odor cues, such as food odors [60]. We therefore modeled an agent that is first presented with a CS+ followed by reward and then is placed in a two-dimensional environment and must navigate to the rewarded odor (Fig 9A, top). The activity of the mushroom body output circuitry controls the forward velocity *u*(*t*) and angular velocity *ω*(*t*) of the agent. The agent’s heading is given by $\frac{d\theta }{dt}=\omega \left(t\right)$, which, along with the forward velocity, determines the change in its location $\frac{dx}{dt}=u\left(t\right)(\cos\theta \left(t\right){\hat{x}}_{1}+\sin\theta \left(t\right){\hat{x}}_{2})$ (Fig 9A). We assumed that these movement variables are not decoded directly from output neurons but from other feedback neurons which may represent locomotion-related downstream regions (see Methods). The environment contains multiple odor sources that produce odor plumes that the the agent encounters as it moves. The mushroom body output circuitry supports this behavior by integrating odor concentration input from Kenyon cells and information from other brain areas about wind direction relative to the agent’s orientation [61] (Fig 9A, bottom; see Methods for a description of how wind input is encoded). Because **x**(*t*) is a differentiable function of network parameters, we can use as a loss function the Euclidean distance between the agent’s location and the rewarded odor source at the end of this navigation period:

$$\begin{array}{c} L_{\theta }=||x\left(T\right)-x^{*}{\left|\right|}^{2}, \end{array}$$ (7)

where x* is the location of the rewarded odor source and *T* is the time at which the navigation period ends. Successfully executing this behavior requires storing the identity of the rewarded odor, identifying the upwind direction for that odor, moving toward the odor source using concentration information, and ignoring neutral odors.

The agent successfully navigates to the rewarded odor source (Fig 9B), and success requires plasticity during conditioning that encodes the CS+/US pairing (S6 Fig). We wondered whether dopamine-gated plasticity might also be operative during navigation, based on recent findings that recorded ongoing dopamine neuron fluctuations correlated with movement [33]. We asked whether such plasticity during navigation affects the behavior of the model by examining the performance of networks in which it is blocked after optimization. Blocking plasticity during navigation impairs performance (Fig 9C). In particular, networks lacking plasticity often exhibit decreased forward velocity after entering a plume corresponding to a rewarded odor (Fig 9B), suggesting that ongoing plasticity may reinforce salient odors as they are encountered and promote odor-seeking, consistent with a recent report [62]. These results make the prediction that increased levels of dopamine neuron activity and dopamine-gated plasticity occur upon encounters of rewarded odor plumes.

We also examined the relationship between dopamine neuron activity and movement variables during navigation. The agent exhibits increased forward velocity and turning upon encountering an odor, with greater increases for rewarded than for neutral odors (Fig 9D). Model dopamine neurons exhibit activity during navigation that correlates with movement (Fig 9E and S7 Fig). Many of the same dopamine neurons also exhibit reward-related activity, as has been observed in neural recordings [33].

An important caveat to our results is that it is possible to construct networks in which plasticity is active during the conditioning phase but gated off during the navigation phase of the task from the beginning of optimization. In natural environments where learning and navigation are not clearly separated into distinct phases, such a gating mechanism may be difficult to implement. However, in our setting, these networks exhibit similar performance to networks in which plasticity is always active (S6 Fig). Thus, unconstrained optimization of networks produces solutions in which ongoing plasticity during navigation is behaviorally relevant (Fig 9B and 9C), but our results cannot be taken to conclude that this plasticity is always required to solve our task. More complex tasks that require moment-by-moment decisions to be made throughout the navigation process may rely on such plasticity and are an exciting direction for future study.

## Discussion

We have developed models of the mushroom body that use a biologically plausible form of dopamine-gated synaptic plasticity to solve a variety of learning tasks. By optimizing the mushroom body output circuitry for task performance, these models generate patterns of dopamine neuron activity sufficient to produce the desired behaviors. Model dopamine responses are distributed, tuned to multiple task-relevant variables, and exhibit rich temporal fluctuations. This diversity is a result of optimizing our models only for task performance rather than assuming that dopamine neurons uniformly represent a particular quantity of interest, such as a global reward prediction error signal [3]. Our results predict that individual dopamine neurons may exhibit diverse tuning while producing coherent activity at the population level. They also provide the first unified modeling framework that can account for valence and reward prediction (Fig 5), novelty (Fig 6), and movement-related (Fig 9) dopamine neuron responses that have been recorded in experiments.

### Relationship to other modeling approaches

To construct our mushroom body models, we took advantage of recent advances in recurrent neural network optimization to augment standard network architectures with dopamine-gated plasticity. Our approach can be viewed as a form of “meta-learning” [45–47], or “learning to learn,” in which a network learns through gradient descent to use a differentiable form of synaptic plasticity (Eq 4) to solve a set of tasks. As we have shown, this meta-learning approach allows us to construct networks that exhibit continual learning and can form associations based on single CS-US pairings (Fig 7). Recent studies have modeled networks with other forms of differentiable plasticity, including Hebbian plasticity, [63–65] but have not studied gated plasticity of the form of Eq 4. Another recent study examined networks with a global neuromodulatory signal rather than the heterogeneous signals we focus on [66]. Meta-learning approaches have also recently been applied to infer alternative learning algorithms to backpropagation through time [67].

Another recent study used a meta-learning approach to model dopamine activity and activity in the prefrontal cortex of mammals [68]. Unlike our study, in which the “slow” optimization is taken to represent evolutionary and developmental processes that determine the mushroom body output circuitry, in this study the slow component of learning involved dopamine-dependent optimization of recurrent connections in prefrontal cortex. This process relied on gradient descent in a recurrent network of long short-term memory (LSTM) units, leaving open the biological implementation of such a learning process. Like in actor-critic models of the basal ganglia [69], dopamine was modeled as a global reward prediction error signal.

In our case, detailed knowledge of the site and functional form of plasticity [28] allowed us to build models that solved multiple tasks using only a biologically plausible synaptic plasticity rule. This constraint allows us to predict patterns of dopamine neuron activity that are sufficient for solving these tasks (Fig 5). Similar approaches may be effective for modeling other brain areas in which the neurons responsible for conveying “error” signals can be identified, such as the cerebellum or basal ganglia [2, 70].

### Heterogeneity of dopamine signaling across species

Dopamine is responsible for a variety of functions in arthropods, including associative memory in honeybees [6], central pattern generation in the stomatogastric ganglion of lobsters [7], escape behaviors [8] and salivation [9] in the cockroach, and flight production in moths [10]. While dopamine similarly plays many roles in *Drosophila*, including the regulation of locomotion, arousal, sleep, and mating [11], until recently most studies of *Drosophila* mushroom body dopamine neurons have focused on their roles in appetitive and aversive memory formation [12, 13, 16, 18, 20–22]. In mammals, while numerous studies have similarly focused on reward prediction error encoding in midbrain dopaminergic neurons [2], recent reports have also described heterogeneity in dopamine signals reminiscent of the heterogeneity across dopamine neurons in the mushroom body [5, 43]. These include reports detailing distinct subtypes of dopamine neurons that convey positive or negative valence signals or respond to salient signals of multiple valences [39, 71], novelty responses [34–38, 40], responses to threat [72], and modulation of dopamine neurons by movement [41, 42]. In many cases, these subtypes are defined by their striatal projection targets, suggesting a compartmentalization of function similar to that of the mushroom body [5]. However, the logic of this compartmentalization is not yet clear.

Standard reinforcement learning models of the basal ganglia, such as actor-critic models, assume that dopamine neurons are globally tuned to reward prediction error signals [69]. Proposals have been made to account for heterogeneous dopamine responses, including that different regions produce prediction errors based on access to distinct state information [73], or that dopamine neurons implement an algorithm for learning the statistics of transitions between states using sensory prediction errors [74]. Our results are compatible with these theories, but different in that our model does not assume a priori that all dopamine neurons encode prediction errors. Instead, prediction error coding by particular modes of population activity emerges in our model as a consequence of optimizing for task performance (Fig 5). This heterogeneity emerged even though we penalized dopamine activity that exceeded a baseline value (Eq 3). In networks in which this penalization is absent, such as networks whose dopamine neurons encode arbitrary changes in internal state (Fig 8), an even higher level of dopamine fluctuations is present in the optimized models.

### Connecting mushroom body architecture and function

The identification of groups of dopamine neurons that respond to positive or negative valence US [16, 24, 30, 75, 76], output neurons whose activity promotes approach or avoidance [26], and dopamine-gated plasticity of KC-to-MBON synapses [27, 28, 77] has led to effective models of first-order appetitive and aversive conditioning in *Drosophila*. A minimal model of such learning requires only two compartments of opposing valence and no recurrence among output neurons or dopamine neurons. The presence of extensive recurrence [33, 48, 52, 78] and dopamine neurons that are modulated by other variables [29, 31–33] suggests that the mushroom body modulates learning and behavior along multiple axes.

The architecture of our model reflects the connectivity between Kenyon cells and output neurons, compartmentalization among output neurons and dopamine neurons, and recurrence of the mushroom body output circuitry. These constraints match the key architectural features of the mushroom body, but also reflect simplifications made in the absence of additional data. While the identities and functional properties of output neurons and dopamine neurons have been mapped anatomically [48, 79], the feedback pathways have not, so the feedback neurons in our model (gray neurons in Fig 1A) represent any neurons that participate in recurrent loops involving the mushroom body, which may involve paths through other brain areas. For most of our analyses (but see S3 Fig), we also neglected direct projections from Kenyon cells to dopamine neurons [49, 56]. When they were added to the model, our qualitative results were unchanged, although it is possible that future studies may uncover a specific role for these connections. Our model could also be extended by including direct depolarizing or hyperpolarizing effects of dopamine on output neurons, which has been observed experimentally [50], or by introducing recurrence among Kenyon cells [49]. Additionally, explicitly modeling the integration of projection neuron to Kenyon cell signaling could provide a more realistic account of the representation of sensory stimuli [80].

Our model could also be extended by considering other forms of synaptic plasticity. There is evidence that dopamine-gated synaptic plasticity rules (Fig 1B) are heterogeneous across compartments [26, 27], and non-dopamine-dependent plasticity could also lead to new behavior [80]. While we have primarily focused on the formation of associations over short timescales because the detailed parameters of compartment-specific learning rules have not been described, such heterogeneity will likely be particularly important in models of long-term memory [21, 81–85].

Our model makes several predictions. It predicts that reward prediction error should emerge as a dominant mode of population activity across dopamine neurons, even though individual dopamine neurons may be diversely tuned (Fig 5). It predicts that compartments that exhibit a large degree of non-specific potentiation may be particularly important for forming short-term associations in complex environments with many background or distractor odors (Fig 7). It also suggests the possibility of pairing an odor presentation with a change in internal state and reading out this pairing from the pattern of output neuron activity upon a subsequent presentation (Fig 8C). Our results also suggest that plasticity during navigation may promote odor-seeking (Fig 9), an idea with experimental support [62]. For each of these predictions, input to dopamine neurons from pathways other than those that convey purely external reinforcement is required. Identifying the pathways that convey these signals is an important direction. In the absence of an explicit correspondence between neurons in our model and their biological counterparts, direct analysis of the connectivity in our optimized networks is unlikely to be sufficient to do so. Future studies should build models that incorporate recently available mushroom body wiring diagrams to further constrain models [49, 50, 52, 53].

However, it is unlikely that purely anatomical information, even at the level of a synaptic wiring diagram, will be sufficient to infer how the mushroom body functions [86]. We have used anatomical information and parametrized synaptic plasticity rules along with hypotheses about which behaviors the mushroom body supports to build “task-optimized” models, related to approaches that have been applied to sensory systems [87]. The success of these approaches for explaining neural data relies on the availability of complex tasks that challenge and constrain the computations performed by the models. Therefore, experiments that probe the axes of fly behavior that the mushroom body supports, including behaviors that cannot be described within the framework of classical conditioning, will be a crucial complement to connectivity mapping efforts as models of this system are refined.

## Methods

### Time discretization

For computational efficiency and ease of training, we assume *τ* in Eq 1 is equal to 1 s and simulate the system with a timestep of Δ*t* = 0.5 s, but our results do not depend strongly on these parameters.

### Optimization

Parameters are optimized using PyTorch (www.pytorch.org) with the RMSprop optimizer [88] and a learning rate of 0.001. The loss to be minimized is described by Eqs 3 and 6 or Eq 7 for networks optimized for conditioning tasks, continuous state representations, or navigation respectively. Optimization is performed over a set number of epochs, each of which consists of a batch of *B* = 30 trials. The loss $L_{\theta }^{\text{tot}}\left(m\right)$ for epoch *m* is the average of the individual losses over each trial in the batch:

$$\begin{array}{c} L_{\theta }^{\text{tot}}\left(m\right)=\frac{1}{B}\sum_{b=1}^{B}L_{\theta }\left(b,m\right), \end{array}$$ (8)

where $L_{\theta }\left(b,m\right)$ represents the loss for *b*th trial drawn on epoch *m*.

All optimized weights are initialized as zero mean Gaussian variables. To initialize **W**_recur_, weights from a neuron belonging to neuron type *X* (where *X* = MBON, DAN, or FBN) have 0 mean and variance equal to $\frac{1}{\sqrt{2}N_{X}}$, where *N*_*X*_ equals the number of neurons of type *X*. For **W**_readout_, the variance is 1/*N*_MBON_ while for **W**_ext_, the variance is 1. Bias parameters are initialized at 0.1. At the beginning of each trial, firing rates are reset to an initial state **r**_0_, with **r**_0_ = 0 for output neurons and 0.1 for dopamine neurons or feedback neurons, to permit these neurons to exhibit low levels of baseline activity.

### Conditioning tasks

For conditioning tasks in which the predicted valence of a conditioned stimulus (CS) is reported (such as first- and second-order conditioning and extinction), each CS is encoded by setting 10% of the entries of **r**_KC_ to 1 and the rest to 0. Unconditioned stimuli (US) are encoded by **r**_ext_ which is equal to (1, 0)^*T*^ when a positive-valence US is present, (0, 1)^*T*^ when a negative-valence US is present, and (0, 0)^*T*^ otherwise. CS and US are presented for 2 s. Tasks are split into 30 s intervals (for example conditioning and test intervals; see Fig 3). Stimulus presentation occurs randomly between 5 s and 15 s within these intervals. Firing rates are reset at the beginning of each interval (e.g. **r**(*t* = 30 s) = **r**_0_), which prevents networks from using persistent activity to maintain associations.

When optimizing networks in Fig 3, random extinction and second-order conditioning trials were drawn. With probability 1/2, CS or US are randomly omitted (and the target valence updated accordingly—e.g., if the US is omitted, the network should not report a nonzero valence upon the second CS presentation; Fig 3B) in order to prevent the networks from overgeneralizing to CS that are not paired with reinforcement. Optimization progressed for 5000 epochs for networks trained to perform extinction and second-order conditioning. For networks trained only for first-order conditioning, (Fig 3E, top; Fig 4), only first-order conditioning trials were drawn, and optimization progressed for 2000 epochs.

Principal components of dopamine neuron activity (Fig 5) were estimated using 50 randomly chosen trials of extinction and second-order conditioning in previously optimized networks. To order dopamine neurons based on their response similarity (Fig 5A), hierarchical clustering was performed using the Euclidean distance between the vector of firing rates corresponding to pairs of dopamine neurons during these trials.

For networks also trained to report stimulus novelty (Fig 6), an additional readout dimension *n*(*t*) that is active for the first presentation of a given CS and inactive otherwise is added. The full network readout is then given by

$$\begin{array}{c} R\left(t\right)=(\begin{array}{c} v(t)\\ n(t) \end{array})=W_{\text{readout}}r_{\text{MBON}}\left(t\right), \end{array}$$ (9)

and the loss equals

$$\begin{array}{c} L_{\theta }=\frac{1}{T}\sum_{n=1}^{T}||R\left(t_{n}\right)-R^{*}\left(t_{n}\right){\left|\right|}^{2}+\frac{\lambda }{T}\sum_{n=1}^{T}\sum_{i=1}^{N_{\text{DAN}}}{\left[r_{i}^{\text{DAN}}\left(t_{n}\right)-0.1\right]}_{+}^{2}. \end{array}$$ (10)

Adding this second readout does not significantly impact the performance of the networks for classical conditioning tasks.

### Networks without dopamine-gated plasticity

For networks without dopamine-gated plasticity, KC-to-MBON synaptic weights were optimized through gradient descent, similar to the weights that determine the output circuitry, and then fixed. The time of CS+ presentation is 5 s, and the second CS presentation occurs at 15 s. Networks were optimized to perform first-order conditioning with positive and negative valence US for a fixed set of stimuli numbering between 2 and 20. On half of the trials, a different CS is presented instead of the second CS+ presentation (Fig 4B) and networks must not respond to this CS.

### Continual learning

To model continual learning (Fig 7), networks are augmented with non-specific potentiation gated by dopamine neuron activity according to Eq 5. The potentiation parameter *β* is compartment-specific and updated through gradient descent. Each parameter is initialized at 0.01 and constrained to be positive.

Trials consist of 200 s intervals, during which two CS+ and two CS- odors are presented randomly. For each CS, the number of presentations in this interval is chosen from a Poisson distribution with a mean of 2 presentations. Unlike other networks, for these networks the values of **W**_KC→MBON_ at the end of one trial are used as the initial condition for the next trial. To prevent weights from saturating early in optimization, the weights at the beginning (*t* = 0) of trial *l* are set equal to:

$$\begin{array}{c} w_{l}\left(0\right)=\left(1-x\right)w_{0}+xw_{l-1}\left(T\right), \end{array}$$ (11)

where *w*_0_ = 0.05 corresponds to the initial weight at the beginning of optimization, *w*_*l*−1_(*T*) are the weights at the end (*t* = *T*) of trial *l* − 1, and *x* increases linearly from 0 to 1 during the first 2500 epochs of optimization. Hence, at the end of the optimization phase, *w*_*l*_(*t*) = *w*_*l*−1_(*T*). Networks were optimized for a total of 5000 epochs.

### Networks that encode changes in state

For networks that encode changes in state (Fig 8), we modified our training protocol to include an additional three-dimensional readout of dopamine neuron activity that encodes the state (at each moment in time, the target is equal to 1 for the corresponding readout dimension and 0 for the others; Eq 6). The external input **r**_ext_ is five-dimensional and signals both state transitions using input pulses of length 2 s and the valence of US as before. The length of time between pulses Δ*T*_state_ is a random variable distributed according to Δ*T*_state_ ∼ 10 s · (1 + Exp(1)). Networks were optimized for 500 epochs.

To test how state-dependent dopamine neuron dynamics affect stimulus encoding, a CS is presented for 2 s, beginning 8 s prior to the second state change of a 300 s trial. Afterward, the same CS is presented for 5 s. This was repeated for 50 CS, and the correlation coefficient between output neuron responses during the second 5 s presentation was calculated (Fig 8C).

### Models of navigation

To model navigation toward a rewarded odor source (Fig 9), a CS+/US pairing is presented at *t* = 2 s in a 20 s training interval with a US strength of $r_{i}^{\text{ext}}=0.1$. This is followed by a 200 s interval during which the model organism navigates in a two-dimensional environment.

During navigation, two odor sources are present, one CS+ and one neutral CS. The sources are randomly placed at *x*_1_ = ±1 m and *x*_2_ chosen uniformly between 0 m and 2 m, with a minimum spacing of 0.5 m. Associated with each odor source is a wind stream that produces an odor plume that the model organism encounters as it navigates. These are assumed to be parallel to the horizontal *x*_1_ axis and oriented so that the odor plume diffuses toward the origin, with a vertical height of 0.5 m and centered on the *x*_2_ position of each odor source. For locations within these plumes and downwind of an odor source, the concentration of the odor is given by:

$$\begin{array}{c} c\left(\Delta x_{1},\Delta x_{2}\right)=\frac{1}{1+0.5\Delta x_{1}}\exp(-{\left(\Delta x_{2}\right)}^{2}/\left(0.1\Delta x_{1}\right)), \end{array}$$ (12)

where Δ*x*_1_ and Δ*x*_2_ are the horizontal and vertical displacements from the odor source in meters. This equation expresses a Gaussian odor plume with a width that increases and magnitude that decreases with distance from the odor source.

During navigation, when the model organism encounters an odor plume, Kenyon cell activity is assumed to be proportional to the pattern of activity evoked by an odor (as before, a random pattern that activates 10% of Kenyon cells) scaled by *c*(Δ*x*_1_, Δ*x*_2_). The network further receives 4-dimensional wind direction input via **W**_ext_ (representing the magnitude in each of the cardinal directions with respect to the model organism). Each input is given by [**w** ⋅ **h**_*i*_]_+_, where **w** is a unit vector representing wind direction and **h**_*i*_ for *i* = 1…4 is a unit vector pointing in the anterior, posterior, or lateral directions with respect to the model organism.

The organism is initially placed at the origin and at an angle distributed uniformly on the range $\left[\frac{\pi }{2}\left(1-\gamma \right),\frac{\pi }{2}\left(1+\gamma \right)\right]$, with *γ* increasing linearly from 0 to 0.5 during the optimization. The movement of the organism is given by two readouts of the feedback neurons. The first determines the forward velocity *u*(*t*) = Softplus(**W**_*u*_ · **r**(*t*) + *b_u_*), and the second determines the angular velocity *ω*(*t*) = **W**_*ω*_ ⋅ **r**(*t*) + *b*_*ω*_. The weights and bias parameters of these readouts are included in the parameter vector *θ* that is optimized using gradient descent. For each trial, the loss is determined by the Euclidean distance of the model organism from the rewarded odor source at the end of the navigation interval (Eq 7). Networks were optimized for 500 epochs. Networks that failed to converge (defined as an average position error of greater than 0.4 m) were discarded.

## Supporting information

S1 Fig Loss function over the course of optimization. Loss is shown for five networks optimized to perform first-order conditioning, second-order conditioning, and extinction (as in Fig 3).
(EPS)

S2 Fig Behavior of networks optimized to perform classical conditioning on a reversal learning task. **(A)** Top: Schematic of reversal learning task. In the first phase, CS1 but not CS2 is paired with US, while during reversal the contingencies are reversed. Preference between CS1 and CS2 is compared in the test phase. Bottom: Example MBON and DAN activity during reversal learning. **(B)** The average difference in reported valence for CS2 vs. CS1. Positive or negative values for positive or negative-valence US, respectively, indicate successful reversal learning. Bars indicate standard deviation across model networks.
(EPS)

S3 Fig Behavior of networks with direct KC-to-DAN connections. Performance on extinction (top) and second-order conditioning (bottom) is shown for control networks (black), networks with optimized KC-to-DAN connections (blue), and networks with KC-to-DAN connections that undergo plasticity according to Eq 4 (green). Corresponding networks that lack recurrence are shown in red, cyan, and magenta, respectively. In all cases, the addition of KC-to-DAN connections does not qualitatively change the results. Error rates for each network realization are evaluated over 50 test trials and used to generate *p*-values with a Mann-Whitney *U*-test over 12 network realizations. For networks in which KC-to-DAN connections are optimized with gradient descent, we initialized the weights uniformly between 0 and 0.05 and constrained them to remain positive during optimization. The optimization procedure reduced the magnitude of these weights significantly across networks in the control (0.025 to 0.021; *p* < 10^−4^) but not no recurrence condition (0.025 to 0.026; n.s.), suggesting that these weights are not critical for performance.
(EPS)

S4 Fig Relationship between US response and associated compartment readout. Results are shown for a representative example optimized network as in Fig 3. Each point represents one compartment. The horizontal axis denotes the corresponding entry of **W**_readout_ and the vertical axis denotes the difference between that compartment’s dopamine neuron’s response to a positive and negative valence US. Consistent with learning involving depression of KC-to-MBON synapses, compartments whose output neuron activity biases the readout toward negative valence (avoidance) have dopamine neurons that preferentially respond to positive valence US.
(EPS)

S5 Fig Performance of networks with fixed numbers of output neurons and dopamine neurons, but different numbers of compartments. For a network with *N*_*c*_ compartments, the dopamine neuron to output neuron connection matrix is block-diagonal with *N*_*c*_ blocks of nonzero weights. Weights within blocks are equal to 1/*N*_*c*_. *N*_*c*_ = 20 corresponds to the networks used elsewhere in this manuscript. Error bars represent s.e.m. across 10 network realizations. Error rates for each network realization are evaluated over 50 test trials and used to generate *p*-values with a Mann-Whitney *U*-test over 10 network realizations. Comparisons are made to networks with *N*_*c*_ = 20.
(EPS)

S6 Fig Dependence of navigation performance on synaptic plasticity. **(A)** Performance of control networks from Fig 9 (black), networks that were optimized with KC-to-MBON plasticity only active during training and not navigation (gray), and networks lacking plasticity altogether (blue). The gray condition corresponds to networks for which plasticity was blocked during optimization and testing, rather than only during testing (red bar in Fig 9C). Error rates for each network realization are evaluated over 50 test trials and used to generate *p*-values with a Mann-Whitney *U*-test over 30 network realizations. **(B)** Similar to Fig 9B, but for a network lacking KC-to-MBON synaptic plasticity altogether. The model organism is unable to identify the rewarded odor and navigate toward it. Trajectories tend toward points located between the two odor sources.
(EPS)

S7 Fig Example cross-correlation functions between dopamine neuron activity and velocity during navigation. Left: Expectation of *d*(*t*)*u*(*t* + *τ*), where *d*(*t*) is dopamine neuron activity and *u*(*t*) is forward velocity. Each color represents a different dopamine neuron. Right: Same as left, but for *d*(*t*)*ω*(*t* + *τ*), where *ω*(*t*) is angular velocity.
(EPS)

## References

[1] Perisse E, Burke C, Huetteroth W, Waddell S. Shocking revelations and saccharin sweetness in the study of Drosophila olfactory memory. Current Biology. 2013;23(17):R752–R763. doi: 10.1016/j.cub.2013.07.060 24028959 PMC3770896

[2] Watabe-Uchida M, Eshel N, Uchida N. Neural Circuitry of Reward Prediction Error. Annual Review of Neuroscience. 2017;40:373–394. doi: 10.1146/annurev-neuro-072116-031109 PMC6721851 28441114

[3] Schultz W, Dayan P, Montague PR. A neural substrate of prediction and reward. Science. 1997;275(5306):1593–1599. doi: 10.1126/science.275.5306.1593 9054347

[4] Sutton RS, Barto AG. Reinforcement Learning: An Introduction. Cambridge, MA: MIT Press; 1998.

[5] Watabe-Uchida M, Uchida N. Multiple Dopamine Systems: Weal and Woe of Dopamine. Cold Spring Harbor Symposia on Quantitative Biology. 2019; p. 037648. 10.1101/sqb.2018.83.037648 30787046

[6] Bicker G, Menzel R. Chemical codes for the control of behaviour in arthropods. Nature. 1989;337(6202):33–39. doi: 10.1038/337033a0 2562906

[7] Marder E, Eisen JS. Electrically coupled pacemaker neurons respond differently to same physiological inputs and neurotransmitters. Journal of Neurophysiology. 1984;51(6):1362–1374. doi: 10.1152/jn.1984.51.6.1362 6145758

[8] Casagrand JL, Ritzmann RE. Biogenic amines modulate synaptic transmission between identified giant interneurons and thoracic interneurons in the escape system of the cockroach. Journal of Neurobiology. 1992;23(6):644–655. doi: 10.1002/neu.480230604 1331317

[9] Evans AM, Green KL. Characterization of the dopamine receptor mediating the hyperpolarization of cockroach salivary gland acinar cells in vitro. British Journal of Pharmacology. 1990;101(1):103–108. doi: 10.1111/j.1476-5381.1990.tb12097.x 2282452 PMC1917631

[10] Claassen DE, Kammer AE. Effects of octopamine, dopamine, and serotonin on production of flight motor output by thoracic ganglia of Manduca sexta. Journal of Neurobiology. 1986;17(1):1–14. doi: 10.1002/neu.480170102 3088211

[11] Yamamoto S, Seto ES. Dopamine Dynamics and Signaling in Drosophila: An Overview of Genes, Drugs and Behavioral Paradigms. Experimental Animals. 2014;63(2):107–119. doi: 10.1538/expanim.63.107 24770636 PMC4160991

[12] Han KA, Millar NS, Grotewiel MS, Davis RL. DAMB, a Novel Dopamine Receptor Expressed Specifically in Drosophila Mushroom Bodies. Neuron. 1996;16(6):1127–1135. doi: 10.1016/S0896-6273(00)80139-7 8663989

[13] de Belle J, Heisenberg M. Associative odor learning in Drosophila abolished by chemical ablation of mushroom bodies. Science. 1994;263(5147):692–695. doi: 10.1126/science.8303280 8303280

[14] Dubnau J, Grady L, Kitamoto T, Tully T. Disruption of neurotransmission in Drosophila mushroom body blocks retrieval but not acquisition of memory. Nature. 2001;411(6836):476–480. doi: 10.1038/35078077 11373680

[15] McGuire SE, Le PT, Davis RL. The Role of Drosophila Mushroom Body Signaling in Olfactory Memory. Science. 2001;293(5533):1330–1333. doi: 10.1126/science.1062622 11397912

[16] Schwaerzel M, Monastirioti M, Scholz H, Friggi-Grelin F, Birman S, Heisenberg M. Dopamine and octopamine differentiate between aversive and appetitive olfactory memories in Drosophila. Journal of Neuroscience. 2003;23(33):10495–10502. doi: 10.1523/JNEUROSCI.23-33-10495.2003 14627633 PMC6740930

[17] Schroll C, Riemensperger T, Bucher D, Ehmer J, Völler T, Erbguth K, et al. Light-Induced Activation of Distinct Modulatory Neurons Triggers Appetitive or Aversive Learning in Drosophila Larvae. Current Biology. 2006;16(17):1741–1747. doi: 10.1016/j.cub.2006.07.023 16950113

[18] Kim YC, Lee HG, Han KA. D1 Dopamine Receptor dDA1 Is Required in the Mushroom Body Neurons for Aversive and Appetitive Learning in Drosophila. Journal of Neuroscience. 2007;27(29):7640–7647. doi: 10.1523/JNEUROSCI.1167-07.2007 17634358 PMC6672866

[19] Claridge-Chang A, Roorda RD, Vrontou E, Sjulson L, Li H, Hirsh J, et al. Writing Memories with Light-Addressable Reinforcement Circuitry. Cell. 2009;139(2):405–415. doi: 10.1016/j.cell.2009.08.034 19837039 PMC3920284

[20] Aso Y, Siwanowicz I, Bräcker L, Ito K, Kitamoto T, Tanimoto H. Specific Dopaminergic Neurons for the Formation of Labile Aversive Memory. Current Biology. 2010;20(16):1445–1451. doi: 10.1016/j.cub.2010.06.048 20637624 PMC2929706

[21] Aso Y, Herb A, Ogueta M, Siwanowicz I, Templier T, Friedrich AB, et al. Three Dopamine Pathways Induce Aversive Odor Memories with Different Stability. PLOS Genetics. 2012;8(7):e1002768. doi: 10.1371/journal.pgen.1002768 22807684 PMC3395599

[22] Burke CJ, Huetteroth W, Owald D, Perisse E, Krashes MJ, Das G, et al. Layered reward signalling through octopamine and dopamine in Drosophila. Nature. 2012;492(7429):433–437. doi: 10.1038/nature11614 23103875 PMC3528794

[23] Aso Y, Sitaraman D, Ichinose T, Kaun KR, Vogt K, Belliart-Guérin G, et al. Mushroom body output neurons encode valence and guide memory-based action selection in Drosophila. eLife. 2014;3:e04580. doi: 10.7554/eLife.04580 25535794 PMC4273436

[24] Owald D, Felsenberg J, Talbot CB, Das G, Perisse E, Huetteroth W, et al. Activity of defined mushroom body output neurons underlies learned olfactory behavior in Drosophila. Neuron. 2015;86(2):417–427. doi: 10.1016/j.neuron.2015.03.025 25864636 PMC4416108

[25] Tanimoto H, Heisenberg M, Gerber B. Event timing turns punishment to reward. Nature. 2004;430(7003):983–983. doi: 10.1038/430983a 15329711

[26] Hige T, Aso Y, Modi MN, Rubin GM, Turner GC. Heterosynaptic Plasticity Underlies Aversive Olfactory Learning in Drosophila. Neuron. 2015;88(5):985–998. doi: 10.1016/j.neuron.2015.11.003 26637800 PMC4674068

[27] Aso Y, Rubin GM. Dopaminergic neurons write and update memories with cell-type-specific rules. eLife. 2016;5:e16135. doi: 10.7554/eLife.16135 27441388 PMC4987137

[28] Handler A, Graham TGW, Cohn R, Morantte I, Siliciano AF, Zeng J, et al. Distinct Dopamine Receptor Pathways Underlie the Temporal Sensitivity of Associative Learning. Cell. 2019;178(1):60–75.e19. doi: 10.1016/j.cell.2019.05.040 31230716 PMC9012144

[29] Hattori D, Aso Y, Swartz KJ, Rubin GM, Abbott LF, Axel R. Representations of Novelty and Familiarity in a Mushroom Body Compartment. Cell. 2017;169(5):956–969.e17. doi: 10.1016/j.cell.2017.04.028 28502772 PMC5806120

[30] Riemensperger T, Völler T, Stock P, Buchner E, Fiala A. Punishment prediction by dopaminergic neurons in Drosophila. Current Biology. 2005;15(21):1953–1960. doi: 10.1016/j.cub.2005.09.042 16271874

[31] Felsenberg J, Barnstedt O, Cognigni P, Lin S, Waddell S. Re-evaluation of learned information in Drosophila. Nature. 2017;544(7649):240–244. doi: 10.1038/nature21716 28379939 PMC5392358

[32] Felsenberg J, Jacob PF, Walker T, Barnstedt O, Edmondson-Stait AJ, Pleijzier MW, et al. Integration of Parallel Opposing Memories Underlies Memory Extinction. Cell. 2018;175(3):709–722. doi: 10.1016/j.cell.2018.08.021 30245010 PMC6198041

[33] Cohn R, Morantte I, Ruta V. Coordinated and compartmentalized neuromodulation shapes sensory processing in Drosophila. Cell. 2015;163(7):1742–1755. doi: 10.1016/j.cell.2015.11.019 26687359 PMC4732734

[34] Steinfels GF, Heym J, Strecker RE, Jacobs BL. Behavioral correlates of dopaminergic unit activity in freely moving cats. Brain Research. 1983;258(2):217–228. doi: 10.1016/0006-8993(83)91145-9 6824912

[35] Ljungberg T, Apicella P, Schultz W. Responses of monkey dopamine neurons during learning of behavioral reactions. Journal of Neurophysiology. 1992;67(1):145–163. doi: 10.1152/jn.1992.67.1.145 1552316

[36] Horvitz JC, Stewart T, Jacobs BL. Burst activity of ventral tegmental dopamine neurons is elicited by sensory stimuli in the awake cat. Brain Research. 1997;759(2):251–258. doi: 10.1016/S0006-8993(97)00265-5 9221945

[37] Rebec GV, Christensen JRC, Guerra C, Bardo MT. Regional and temporal differences in real-time dopamine efflux in the nucleus accumbens during free-choice novelty. Brain Research. 1997;776(1):61–67. doi: 10.1016/S0006-8993(97)01004-4 9439796

[38] Lak A, Stauffer WR, Schultz W. Dopamine neurons learn relative chosen value from probabilistic rewards. eLife. 2016;5:e18044. doi: 10.7554/eLife.18044 27787196 PMC5116238

[39] Bromberg-Martin ES, Matsumoto M, Hikosaka O. Dopamine in Motivational Control: Rewarding, Aversive, and Alerting. Neuron. 2010;68(5):815–834. doi: 10.1016/j.neuron.2010.11.022 21144997 PMC3032992

[40] Menegas W, Babayan BM, Uchida N, Watabe-Uchida M. Opposite initialization to novel cues in dopamine signaling in ventral and posterior striatum in mice. eLife. 2017;6:e21886. doi: 10.7554/eLife.21886 28054919 PMC5271609

[41] Howe MW, Dombeck DA. Rapid signalling in distinct dopaminergic axons during locomotion and reward. Nature. 2016;535(7613):505–510. doi: 10.1038/nature18942 27398617 PMC4970879

[42] Engelhard B, Finkelstein J, Cox J, Fleming W, Jang HJ, Ornelas S, et al. Specialized coding of sensory, motor and cognitive variables in VTA dopamine neurons. Nature. 2019;570(7762):509. doi: 10.1038/s41586-019-1261-9 31142844 PMC7147811

[43] Cox J, Witten IB. Striatal circuits for reward learning and decision-making. Nature Reviews Neuroscience. 2019;20(8):482–494. doi: 10.1038/s41583-019-0189-2 31171839 PMC7231228

[44] Sussillo D, Abbott LF. Generating coherent patterns of activity from chaotic neural networks. Neuron. 2009;63(4):544–557. doi: 10.1016/j.neuron.2009.07.018 19709635 PMC2756108

[45] Duan Y, Schulman J, Chen X, Bartlett PL, Sutskever I, Abbeel P. RL^2^: Fast Reinforcement Learning via Slow Reinforcement Learning. arXiv. 2016;1611.02779.

[46] Finn C, Abbeel P, Levine S. Model-agnostic Meta-learning for Fast Adaptation of Deep Networks. In: Proceedings of the 34th International Conference on Machine Learning. vol. 70; 2017. p. 1126–1135.

[47] Wang JX, Kurth-Nelson Z, Tirumala D, Soyer H, Leibo JZ, Munos R, et al. Learning to reinforcement learn. arXiv. 2016;1611.05763.

[48] Aso Y, Hattori D, Yu Y, Johnston RM, Iyer NA, Ngo TT, et al. The neuronal architecture of the mushroom body provides a logic for associative learning. eLife. 2014;3:e04577. doi: 10.7554/eLife.04577 25535793 PMC4273437

[49] Eichler K, Li F, Litwin-Kumar A, Park Y, Andrade I, Schneider-Mizell CM, et al. The complete connectome of a learning and memory centre in an insect brain. Nature. 2017;548(7666):175–182. doi: 10.1038/nature23455 28796202 PMC5806122

[50] Takemura S, Aso Y, Hige T, Wong A, Lu Z, Xu CS, et al. A connectome of a learning and memory center in the adult Drosophila brain. eLife. 2017;6:e26975. doi: 10.7554/eLife.26975 28718765 PMC5550281

[51] Zheng Z, Lauritzen JS, Perlman E, Robinson CG, Nichols M, Milkie D, et al. A Complete Electron Microscopy Volume of the Brain of Adult Drosophila melanogaster. Cell. 2018;174(3):730–743. doi: 10.1016/j.cell.2018.06.019 30033368 PMC6063995

[52] Eschbach C, Fushiki A, Winding M, Schneider-Mizell CM, Shao M, Arruda R, et al. Recurrent architecture for adaptive regulation of learning in the insect brain. Nature Neuroscience. 2020;23(4):544–555. doi: 10.1038/s41593-020-0607-9 32203499 PMC7145459

[53] Li F, Lindsey JW, Marin EC, Otto N, Dreher M, Dempsey G, et al. The connectome of the adult Drosophila mushroom body provides insights into function. eLife. 2021;10:e67510. 33315010 10.7554/eLife.62576 PMC7909955

[54] Kirkpatrick J, Pascanu R, Rabinowitz N, Veness J, Desjardins G, Rusu AA, et al. Overcoming catastrophic forgetting in neural networks. Proceedings of the National Academy of Sciences. 2017;114(13):3521–3526. doi: 10.1073/pnas.1611835114 28292907 PMC5380101

[55] Zador AM. A critique of pure learning and what artificial neural networks can learn from animal brains. Nature Communications. 2019;10(1):3770. doi: 10.1038/s41467-019-11786-6 PMC6704116 31434893

[56] Cervantes-Sandoval I, Phan A, Chakraborty M, Davis RL. Reciprocal synapses between mushroom body and dopamine neurons form a positive feedback loop required for learning. eLife. 2017;6:e23789. doi: 10.7554/eLife.23789 28489528 PMC5425253

[57] Hopfield JJ. Neural networks and physical systems with emergent collective computational abilities. Proceedings of the National Academy of Sciences. 1982;79(8):2554–2558. doi: 10.1073/pnas.79.8.2554 PMC346238 6953413

[58] Fusi S, Abbott LF. Limits on the memory storage capacity of bounded synapses. Nature Neuroscience. 2007;10(4):485–493. doi: 10.1038/nn1859 17351638

[59] Krashes MJ, DasGupta S, Vreede A, White B, Armstrong JD, Waddell S. A Neural Circuit Mechanism Integrating Motivational State with Memory Expression in Drosophila. Cell. 2009;139(2):416–427. doi: 10.1016/j.cell.2009.08.035 19837040 PMC2780032

[60] Gaudry Q, Nagel KI, Wilson RI. Smelling on the fly: sensory cues and strategies for olfactory navigation in Drosophila. Current Opinion in Neurobiology. 2012;22(2):216–222. doi: 10.1016/j.conb.2011.12.010 22221864 PMC3323672

[61] Suver MP, Matheson AMM, Sarkar S, Damiata M, Schoppik D, Nagel KI. Encoding of Wind Direction by Central Neurons in Drosophila. Neuron. 2019;102(4):828–842.e7. doi: 10.1016/j.neuron.2019.03.012 30948249 PMC6533146

[62] Sayin S, De Backer JF, Siju KP, Wosniack ME, Lewis LP, Frisch LM, et al. A Neural Circuit Arbitrates between Persistence and Withdrawal in Hungry Drosophila. Neuron. 2019;104(3):544–558. doi: 10.1016/j.neuron.2019.07.028 31471123 PMC6839618

[63] Ba J, Hinton GE, Mnih V, Leibo JZ, Ionescu C. Using Fast Weights to Attend to the Recent Past. In: Advances in Neural Information Processing Systems. vol. 29; 2016. p. 4331–4339.

[64] Miconi T, Clune J, Stanley KO. Differentiable plasticity: training plastic neural networks with backpropagation. In: Proceedings of Machine Learning Research. vol. 80; 2018. p. 3559–3568.

[65] Orhan AE, Ma WJ. A diverse range of factors affect the nature of neural representations underlying short-term memory. Nature Neuroscience. 2019;22(2):275. doi: 10.1038/s41593-018-0314-y 30664767

[66] Miconi T, Rawal A, Clune J, Stanley KO. Backpropamine: training self-modifying neural networks with differentiable neuromodulated plasticity. In: International Conference on Learning Representations. 2019.

[67] Bellec G, Scherr F, Subramoney A, Hajek E, Salaj D, Legenstein R, et al. A solution to the learning dilemma for recurrent networks of spiking neurons. Nature Communications 2019;11(1):3625. doi: 10.1038/s41467-020-17236-y PMC7367848 32681001

[68] Wang JX, Kurth-Nelson Z, Kumaran D, Tirumala D, Soyer H, Leibo JZ, et al. Prefrontal cortex as a meta-reinforcement learning system. Nature Neuroscience. 2018;21(6):860–868. doi: 10.1038/s41593-018-0147-8 29760527

[69] Barto AG. Adaptive critics and the basal ganglia. In: Models of information processing in the basal ganglia. Computational neuroscience. Cambridge, MA: The MIT Press; 1995. p. 215–232.

[70] Ito M, Sakurai M, Tongroach P. Climbing fibre induced depression of both mossy fibre responsiveness and glutamate sensitivity of cerebellar Purkinje cells. Journal of Physiology. 1982;324:113–134. doi: 10.1113/jphysiol.1982.sp014103 PMC1250696 7097592

[71] Matsumoto M, Hikosaka O. Two types of dopamine neuron distinctly convey positive and negative motivational signals. Nature. 2009;459(7248):837–841. doi: 10.1038/nature08028 19448610 PMC2739096

[72] Menegas W, Akiti K, Amo R, Uchida N, Watabe-Uchida M. Dopamine neurons projecting to the posterior striatum reinforce avoidance of threatening stimuli. Nature Neuroscience. 2018;21(10):1421–1430. doi: 10.1038/s41593-018-0222-1 30177795 PMC6160326

[73] Lau B, Monteiro T, Paton JJ. The many worlds hypothesis of dopamine prediction error: implications of a parallel circuit architecture in the basal ganglia. Current Opinion in Neurobiology. 2017;46:241–247. doi: 10.1016/j.conb.2017.08.015 28985550

[74] Gardner MPH, Schoenbaum G, Gershman SJ. Rethinking dopamine as generalized prediction error. Proceedings Biological Sciences. 2018;285 (1891). doi: 10.1098/rspb.2018.1645 30464063 PMC6253385

[75] Mao Z, Davis RL. Eight different types of dopaminergic neurons innervate the Drosophila mushroom body neuropil: anatomical and physiological heterogeneity. Frontiers in Neural Circuits. 2009;3:5. doi: 10.3389/neuro.04.005.2009 19597562 PMC2708966

[76] Liu C, Plaçais PY, Yamagata N, Pfeiffer BD, Aso Y, Friedrich AB, et al. A subset of dopamine neurons signals reward for odour memory in Drosophila. Nature. 2012;488(7412):512–516. doi: 10.1038/nature11304 22810589

[77] Berry JA, Phan A, Davis RL. Dopamine Neurons Mediate Learning and Forgetting through Bidirectional Modulation of a Memory Trace. Cell Reports. 2018;25(3):651–662.e5. doi: 10.1016/j.celrep.2018.09.051 30332645 PMC6239218

[78] Ichinose T, Aso Y, Yamagata N, Abe A, Rubin GM, Tanimoto H. Reward signal in a recurrent circuit drives appetitive long-term memory formation. eLife. 2015;4:e10719. doi: 10.7554/eLife.10719 26573957 PMC4643015

[79] Tanaka NK, Tanimoto H, Ito K. Neuronal assemblies of the Drosophila mushroom body. Journal of Comparative Neurology. 2008;508(5):711–755. doi: 10.1002/cne.21692 18395827

[80] Kennedy A. Learning with naturalistic odor representations in a dynamic model of the Drosophila olfactory system. bioRxiv. 2019;783191.

[81] Tully T, Preat T, Boynton SC, Del Vecchio M. Genetic dissection of consolidated memory in Drosophila. Cell. 1994;79(1):35–47. doi: 10.1016/0092-8674(94)90398-0 7923375

[82] Trannoy S, Redt-Clouet C, Dura JM, Preat T. Parallel processing of appetitive short- and long-term memories in Drosophila. Current Biology. 2011;21(19):1647–1653. doi: 10.1016/j.cub.2011.08.032 21962716

[83] Cervantes-Sandoval I, Martin-Peña A, Berry JA, Davis RL. System-like consolidation of olfactory memories in Drosophila. Journal of Neuroscience. 2013;33(23):9846–9854. doi: 10.1523/JNEUROSCI.0451-13.2013 23739981 PMC3733538

[84] Pai TP, Chen CC, Lin HH, Chin AL, Lai JSY, Lee PT, et al. Drosophila ORB protein in two mushroom body output neurons is necessary for long-term memory formation. Proceedings of the National Academy of Sciences. 2013;110(19):7898–7903. doi: 10.1073/pnas.1216336110 23610406 PMC3651462

[85] Aso Y, Ray RP, Long X, Bushey D, Cichewicz K, Ngo TT, et al. Nitric oxide acts as a cotransmitter in a subset of dopaminergic neurons to diversify memory dynamics. eLife. 2019;8:e49257. doi: 10.7554/eLife.49257 31724947 PMC6948953

[86] Bargmann CI, Marder E. From the connectome to brain function. Nature Methods. 2013;10(6):483–490. doi: 10.1038/nmeth.2451 23866325

[87] Yamins DLK, DiCarlo JJ. Using goal-driven deep learning models to understand sensory cortex. Nature Neuroscience. 2016;19(3):356–365. doi: 10.1038/nn.4244 26906502

[88] Tieleman T, Hinton G. Lecture 6.5—RMSProp: Divide the gradient by a running average of its recent magnitude. COURSERA: Neural Networks for Machine Learning. 2012;4(2).

